# Support vector machines for explaining physiological stress response in Wood mice (*Apodemus sylvaticus*)

**DOI:** 10.1038/s41598-018-20646-0

**Published:** 2018-02-07

**Authors:** Beatriz Sánchez-González, Isabel Barja, Ana Piñeiro, M. Carmen Hernández-González, Gema Silván, Juan Carlos Illera, Roberto Latorre

**Affiliations:** 1 Departamento de Biología, Unidad de Zoología, Universidad Autónoma de Madrid, c/Darwin 2, Campus Universitario de Cantoblanco, 28049 Madrid, Spain; 2 Escuela de Medicina Veterinaria, Facultad de Ecología y Recursos Naturales, Universidad Andrés Bello, Santiago de Chile, Chile; 3 Departamento de Fisiología (Fisiología Animal), Facultad de Veterinaria, Universidad Complutense de Madrid, Madrid, Spain; 4 Departamento de Ingeniería Informática, Universidad Autónoma de Madrid, 28049 Madrid, Spain

## Abstract

Physiological stress response is a crucial adaptive mechanism for prey species survival. This paper aims to identify the main environmental and/or individual factors better explaining the stress response in Wood mice, *Apodemus sylvaticus*. We analyzed alterations in fecal glucocorticoid metabolite (FCM) concentration – extensively used as an accurate measure of the physiological stress response – of wild mice fecal samples seasonally collected during three years. Then, support vector machines were built to predict said concentration according to different stressors. These statistical tools appear to be particularly suitable for small datasets with substantial number of dimensions, corroborating that the stress response is an extremely complex process in which multiple factors can simultaneously partake in a context-dependent manner, i.e., the role of each potential stressor varies in time depending on other stressors. However, air-humidity, temperature and body-weight allowed us to explain the FCM fluctuation in 98% of our samples. The relevance of air-humidity and temperature altering FCM level could be linked to the presence of an abundant vegetation cover and, therefore, to food availability and predation risk perception. Body-weight might be related to the stress produced by reproduction and other intraspecific relationships such as social dominance or territorial behavior.

## Introduction

Life forms are influenced by an ever-changing environment. Consequently, animals have developed a wide variety of physiological, behavioral and morphological adaptations to endure harsh conditions and threats^1,2^. Among this extensive array of responses, physiological changes are certainly important, as they increase the energy available for individuals to cope with environmental stressors^3^.

Physiological stress response plays a key role in the adaptability of animals to changes in the environment, as well as being a decisive factor in the stability of homeostasis^4^. The endocrine response begins with the perception of a stressor, which triggers the activation of the hypothalamic-pituitary-adrenocortical (HPA) axis, stimulating the secretion of glucocorticoids (GCs) in the adrenal cortex to overcome adverse situations more effectively^5,6^. The short-term release of GCs is an adaptive response that redirects energy from non-vital activities toward survival^5,7^. However, chronically elevated GC levels due to prolonged exposure to stressors may induce deleterious effects such as reproductive disruption, suppression of the immune function or inhibition of growth, which can lead to a survival rate and fitness reduction^4,5,8^. GCs have been used as indicators of stress in several species including small mammals (e.g., see refs^9–13^). Fecal cortisol/corticosterone metabolites (FCM) reflect free GCs in plasma, yielding an accurate profile of the adrenocortical activity^1,14,15^. Thus, the FCM concentration has been widely used as a suitable non-invasive measure of the GC levels in order to evaluate responses during stressful circumstances, particularly in wildlife studies^16^.

Multiple factors, such as human disturbances^13,17–19^, predator presence^12,13,20^, reproduction^21,22^, social dominance^23,24^ or habitat type and seasonality^1,25,26^, have been reported to influence the mammals physiological stress response in different ways. Rodent populations often show important seasonal and inter-annual fluctuations, decreasing during seasons with unfavorable climatic conditions and increasing during favorable periods^27–29^. Seasonal climatic variations determine food availability and plant abundance, which is crucial for predator avoidance^30,31^. In general, small mammals usually show a strong preference for habitats with a highly dense vegetation cover^32,33^. This increases the survival probability during unfavorable climatic periods and constitutes an effective anti-predator strategy that reduces the exposure to threats^30,31,34–37^. Moonlight can make animals more visible to predators and has a demonstrated effect on small mammals^12,38^. Predation risk does not only induce behavioral changes, but also increases GC secretion as part of the stress response^39^. These vital behavioral and physiological changes have some costs^40^, and prey species should balance daily activities in relation to the predation risk perceived in each moment^41–43^. In the particular case of wood mice (*Apodemus sylvaticus,* Linnaeus, 1758), animals show predilection for evergreen forest and Mediterranean shrub habitats, which provide diverse trophic resources and a large number of shelters due to abundant vegetation cover^29^. Individual characteristics of each animal, such as body condition, sex or breeding condition, can determine significant aspects of the mice biology, e.g., home range^44–46^ or stress level^12,13,47^. In this regard, a relevant result for our study is that the basal GC level of males and females is different due to differences in the GC metabolism and excretion rate^47,48^.

In this paper, we assess the effect of different environmental and individual factors capable of triggering the physiological stress response of wild wood mice. Our goal is to identify which of these factors could most significantly affect the GC level, and hence be the principal stressors in *A*. *sylvaticus*. Taking into account the above-mentioned premises and the properties of the study area, we analyze the relationship between FCM concentrations and the following factors: year, month, season, rainfall, temperature, relative air humidity, habitat, moon phase, sex, breeding condition and body weight. We consider year, season and month to be time periods grouping a set of factors that could produce together a characteristic effect in the stress response (e.g., favorable/unfavorable weather conditions or reproductive period). We hypothesize that abundant rainfall, high humidity and warm temperatures would favor an increase in food availability and, therefore, a smaller physiological stress response. We argue that a higher predation risk perception would elevate the animals’ stress response during adverse climatic periods and in habitats with a reduced vegetation cover. Similarly, full moon would elevate GC levels due to a higher exposure of the animals during bright nights. We also hypothesize increasing GC levels in breeders, due to a greater energy demand, and in juveniles, which are frequently displaced to poorer quality home ranges with less vegetation cover and higher predation risk. Finally, taking into account the strong correlation between weight and body condition, we suppose higher GC levels in smallest and weakest animals due to intraspecific competitions and a poorer defense system against predators.

## Materials and Methods

### Study area

Fieldwork was carried out in Montes do Invernadeiro Natural Park, a protected area of 5,722 ha located in the northwest of the Iberian Peninsula (Spain). Vegetation is mainly constituted by extensive reforestation of Scots pine (*Pinus sylvestris*) and maritime pine (*Pinus pinaster*), and associations of English oak (*Quercus robur*), Pyrenean oak (*Quercus pyrenaica*), white birch (*Betula celtiberica*) and English holly (*Ilex aquifolium*) in valleys and riverbanks. Spanish heath (*Erica australis*), prickled broom (*Pterospartum tridentatum*) and yellow rockrose (*Halimium lasianthum*) are the most representative scrubland species.

Wood mice is the most abundant small mammal species in the study area, but edible dormouse (*Glis glis*), garden dormouse (*Eliomys quercinus*), pygmy shrew (*Sorex minutus*), white-toothed shrew (*Crocidura russula*) and several species of voles, such as European snow vole (*Chionomys nivalis*), are also abundant.

### Live-trapping and data collection

The project had the permission of Montes do Invernadeiro Natural Park (Spain) and regional government of Galicia. All procedures were performed in accordance with the ethical standards of the institution (Universidad Autónoma de Madrid) and in agreement with the Spanish national legislation. Live-trapping was performed seasonally (winter: January-February; spring: June; summer: August; and autumn: October-November) during three consecutive years and in the three main habitats of the study area: pine reforestation, deciduous forest and scrubland. The study area was divided into 9 plots (3 per habitat) separated 3 km to avoid possible replication. In each plot, we placed 25 Sherman live traps separated 10 m each and shaping a 5 × 5 grid. Inside traps, we used 20 g of bread soaked in rancid oil as bait. Traps were oriented against the slope to allow a correct closing. They were covered with vegetation to protect trapped animals, and partially filled with waterproof cotton wool to improve thermal insulation. Traps remained on the field 9 consecutive nights (3 nights per habitat), making check-ups at dawn and dusk to minimize the time animals were captive and to avoid bias in GC levels.

Captured animals were identified by external morphology. Sex and breeding condition were determined according to Gurnell and Flowerdew^49^. Thus, males presented a longer anal-genital distance. Breeding females showed prominent nipples on abdomen and thorax, and vaginal membrane perforated. In breeding adult males, testicles became bigger and marked in the scrotal sac. Individual body weight was measured employing a 100-g hand-held scale (PESNET, 100 g).

We used harmless waterproof paints (Marking stick DFV, www.divasa-farmavic.com) to identify possible recaptures. We marked the animals on paws, inner ear area and tail, where marks were less likely to degrade due to hair loss and they might remain from one season to another. The high mortality in the study area during winter reduced the number of animals recaptured in winter and spring. Animals were more likely recaptured during summer and autumn, identifying them as re-sampled animals. Only new individuals were included in our study to avoid pseudo-replication. After handling, captured individuals were released as fast as possible in the same capture area.

Moon phase was recorded during the night before trap review, considering the percentage of night clouds. Weather conditions – i.e., rainfall, temperature and relative air humidity – were obtained from a weather station located in the study area.

### Fecal samples

Traps were checked twice daily with 12 hours in between review. GC concentration peaks in feces of related mouse species appear around 8–12 hours after the ACTH injection^47,50,51^. Therefore, as wood mice are more active 2–4 hours after sunset^52^, feces of the few individuals trapped during the afternoon were discarded to avoid bias due to any trapping effect on the physiological stress response. Thus, we only included in our study fresh feces, i.e., not dried and with a soft texture^19^, from individuals trapped during the night. In this way, we made sure that the fecal samples analyzed belonged to individuals trapped less than 8 hours. They were collected between sunrise and two hours thereafter to minimize exposure to environmental conditions and microbial action^16,53,54^ and avoid the influence of circadian rhythm in excretion patterns^47,50^. Additionally, we did not take into consideration the traps where urine was detected in order to avoid cross contamination of fecal samples.

Feces in the field were collected in Eppendorf tubes and immediately placed in a portable cooler at 4 °C. After field work, samples were taken to the laboratory and maintained in storage at −20 °C until assayed.

### Extraction and quantification of fecal corticosterone metabolites

Frozen fecal samples were dried at 50 °C during 6 hours in a laboratory heater (Selecta, model CONTERM 2000208) until constant weight. The extraction of GC metabolites from the feces was performed according to Touma *et al*.^47^. Briefly, samples were homogenized with mortar and pestle. Then, 0.05 g were mixed with 1 ml of 80% methanol in an Eppendorf tube. Samples were shaken for 30 minutes on a multivortex and then centrifuged for 15 minutes at 2,500 × g. Supernatants were obtained diluted 1:10 with assay buffer and maintained at −20 °C until analysis.

To analyze the FCM concentration in the extracts, we used an enzyme immunoassay (EIA) developed and validated specifically for wood mice (see below) in the laboratory of Endocrinology of the Veterinary Faculty (Universidad Complutense, Madrid) following the methods described in refs^55–57^. Polyclonal antibody (CT1098) was raised in our laboratory in rabbits against corticosterone 3-CMO: BSA (Steraloids Inc., Newport, USA). Cross reactivity of the corticosterone antibody CT1098 was: Corticosterone: 100%; Aldosterone: 10.5%; Prednisolone: 5.71%; Prednisone: 8.9%; Cortisone: 10.8%; Cortisol: 6.4%; 11-Deoxycorticosterone: 14.31%; 21-Deoxycorticosterone: 5.31%; Progesterone, estradiol, testosterone and estrone sulphate <0.1%. The low detection limit of the assay tested as defined by Abraham^58^ and Munro and Lasley^59^, and calculated from B0 values (maximum binding) minus 2SD in 10 consecutive assays was 3 ng corticosterone metabolites/g dry feces. The addition of exogenous quantities of corticosterone (30.0; 300.0; 3,000.0 ng corticosterone/g feces) to pooled fecal samples with high (23,568.05 ng corticosterone/g feces) and low (534.13 ng corticosterone/g feces) FCM concentrations showed a mean recovery of 98.96% (being 96.50% and 101.5% the minimum and maximum recovery value, respectively) for high FCM samples, and 97.33% (with a minimum and a maximum recovery equal to 95.70% and 99.50%) for low FCM samples. Intra-assay coefficient of variation (CV) was calculated by assaying ten times pools of fecal samples within an assay. Inter-assay CV was calculated by assaying the same pools of fecal samples in ten consecutive assays. Intra- and inter-assay CV were 6.5% and 10.5% in low concentrations, and 5.1% and 9.9% in high FCM concentrations. Parallelism was performed by comparing serial dilutions of pooled fecal extracts and the standard curve demonstrating that binding inhibition curves of serially diluted pools of fecal extracts were parallel to the standard curve (Line formula of standard dose response curve: *Y* = 1.78 − 0.00010*X* and line formula of serial dilutions of pooled data, samples: *Y* = 1.75 − 0.00010*X*, *P* = 0.92, *R*2 = 0.91). FCM concentrations are expressed as ng/g of dry feces.

To confirm suitability of the EIA for monitoring FCM concentrations in wood mice, we performed a physiological validation based on an ACTH stimulation test, which is considered the best test to evaluate adrenal gland functionality^60^. Following the procedure described in Touma *et al*.^50^, we injected a high dose 60 *μ*g/100 g of body weight of synthetic ACTH (Synacthen Depot, Novartis, Germany) into five captive individuals (two females and three males). Samples of each of these individuals were collected within minutes after defecation and immediately stored in Eppendorf tubes at −20 °C until analysis. Sampling times were 0, 2, 4, 6, 8, 10, 12, 14, 18, 22 and 26 hours post injection. Measured FCM baseline levels (prior to injection) for each tested individual ranged from 13,120 to 40,420 ng/g dry feces. In the five individuals, the corticosterone EIA detected an average increase in the FCM concentration in the steroid extracts ranging from 116% to 247% within 8 to 12 h of the ACTH injection. Following, a downward trend towards baseline FCM values was detected within 12 to 18 h, validating the corticosterone EIA for the mouse fecal samples analysis.

### Statistical analysis

#### Support Vector Machines

We employed *support vector machines* (SVMs) to analyze which environmental and individual factors allowed us to explain the physiological stress response of the fecal samples collected during fieldwork. SVMs are supervised statistical learning methods applicable to pattern classification and regression tasks^61–67^. They have captured the attention of the scientific community as they have proven to be powerful and useful tools in a wide range of problems in different disciplines. In particular, SVM-based analyses have been successfully applied to different biological data in areas like environmental science, neuroscience, bioinformatics and medical diagnosis between others^68–73^. Before describing our approach, in this section we provide a brief explanation of what a SVM classifier is.

SVM classifiers are machine-learning tools built to predict the class or category to which a particular object belongs as a function of an *n*-dimensional feature vector (*χ*). They are constructed adjusting by training the parameters of a classification function (Eq. 1) to get an optimal classification of a series of known feature vectors with their corresponding classes. The goal of the training phase is finding a hyperplane in the feature space separating the target classes, which provides the SVM model with the ability to generalize and predict the class of out-of-training samples. Obviously, if the training set does not contain representative samples with relevant information, no empirical model can be constructed.

A SVM classification function is as follows:

1 $$f(\chi )=\sum _{i=1}^{N}\,{\alpha }_{i}\,{y}_{i}\,K(\chi ,{\chi }_{i})+b$$

where ${\{({\chi }_{i},{y}_{i})\}}_{i=1}^{N}$ is the training set with *N* feature vectors (*χ*_*i*_) and their corresponding class (*y*_*i*_); *α*_*i*_ and *b* are parameters to adjust during the training phase; and *K*(*χ*, *χ*_*i*_) is the so-called *kernel function*. A kernel function receives two feature vectors as input and returns a single scalar value measuring the similarity between these vectors. This function is the base of the SVM learning method, since it implicitly maps original input data into a high-dimensional space where the margins separating the data are maximized^64,66^. Different suitable kernel functions can be used in a SVM depending on the problem properties and the distribution of the samples to classify – e.g., polynomial, quadratic or sigmoid kernels^61,65,66^. In this regard, an interesting feature of SVM-based classifiers is their ability to generate linear or nonlinear decision boundaries depending on the kernel function.

#### SVM analysis

Linear SVMs are fast to train and execute. However, the assumption that the data are linearly separable is rarely fulfilled. In contrast, nonlinear SVM analysis provides better performance in many problems, but it loses explanatory capacity as nonlinear kernels map the data to a high-dimensional feature space where it is difficult to interpret the relevance of each original feature. Given the nature of our analysis, to identify which factors were the main stressors of the 105 mice trapped during fieldwork, we carried out what we called a *brute force analysis*. We constructed SVM models based on different kernel functions to predict the stress response according to all the possible combinations of potential stressors, i.e., 1-to-11-dimensional classifiers combining year, month, season, rainfall, temperature, relative air humidity, habitat, moon phase, sex, breeding condition and body weight. Every classifier was trained and tested on all the samples in the dataset. Then, we calculated the percentage of correct predictions in the tests. Analyzing performance of each SVM model, we were able to identify the combinations of environmental and/or individual factors providing a better predictive accuracy and, therefore, the factors that best explained the stress response in our mice fecal samples.

Results reported in this paper correspond to SVM classifiers based on a Gaussian^65,74^ and a linear kernel (Table 1). Gaussian SVMs were the classifiers with a better performance regardless of the factors in the feature vector, while results of the linear SVMs are presented to compare performance of linear and nonlinear analysis.

**Table 1:** Definition of the Gaussian and the linear kernel function.

Kernel function	
Gaussian kernel	${K}_{{\rm{\sigma }}}^{G}(\chi ,{\chi }_{i})={e}^{-{\Vert \chi -{\chi }_{i}\Vert }^{2}/2{{\rm{\sigma }}}^{2}}$
Linear kernel	*K*^*L*^(*χ*, *χ*_*i*_) = *χ* · *χ*_*i*_

#### Categorization of fecal samples

**Figure 1:**
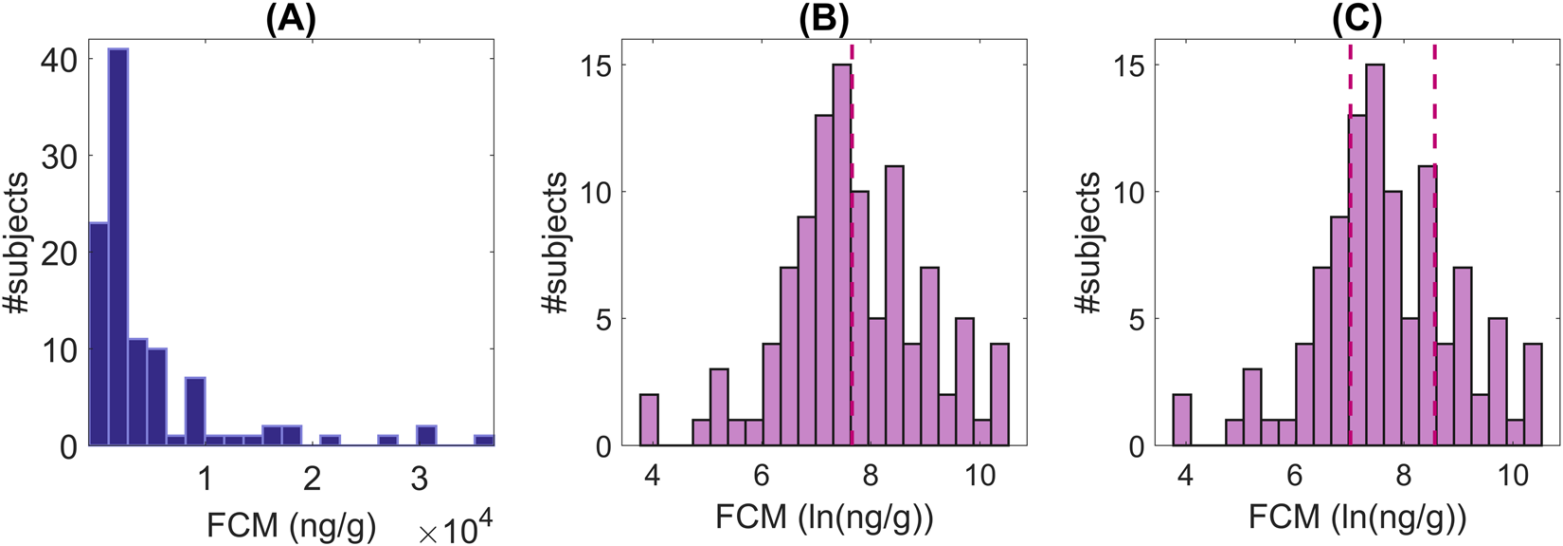
FCM level histogram in linear (**A**) and logarithmic scale (**B**,**C**). Dashed line in panel (B) denotes the threshold between the two categories considered in the two-class categorization. Fecal samples below this threshold are considered to correspond to animals with a low level of stress, while those above this value to animals with a high level of stress. Similarly, dash lines in panel (C) correspond to thresholds in the three-class categorization (low, moderate and high stress level).

Experimental evidence support that physiological stress response is related to the FCM level. We thus assumed that mice could be categorize according to this value in order to study the relationship between occurrence of stress and different potential stressors. This categorization was made theoretically from the FCM level histogram (Fig. 1) as, to the best of our knowledge, there is no experimental evidence regarding specific threshold values determining different levels of stress as a function of the FCM concentration. Taking this into account, we considered that the most stressed wood mice were those with comparatively higher FCM concentrations.

We contemplated two different scenarios. In a first group of experiments, we considered two possible classes: animals with a low and a high level of stress. Fecal samples were distributed according to the FCM level using a 50–50 splitting strategy, i.e., 50% of the samples belonged to each class (Fig. 1B). In a second group of experiments, we categorized the samples in three classes (low, moderate and high) using a 25-50-25 splitting strategy (Fig. 1C). To address this multi-class problem, we used a one-against-all strategy^63^. We trained three different classifiers to separate each class of the other two classes, and we classified out-of-training samples according to the classifier giving the largest output. Results presented in this paper correspond to the three-class distribution, but the ones obtained with the two-class distribution were equivalent.

#### Validation of the SVM classifiers

One of the major problems of SVM learning methods is incurring in overfitting^75^, i.e., even though all feature vectors in the training set might be well classified, the SVM might lose its ability to generalize out of the training set. This problem for generalization was particularly tricky in an analysis like the brute-force analysis proposed here, where the training set was also used as testing set. Then, we validated our SVM models using a 10-fold cross-validation scheme^64^. Fecal samples were randomly divided into ten mutually exclusive subsets of the same size. Nine of these subsets were used as training set to build the model, while the remaining subset was used as testing set to determine the generalization ability of the SVM. We repeated ten times these steps until every subset was used as testing set. The predictive accuracy of the ten tests in the 10-fold cross-validation allowed us to validate whether the SVM model was incurring in overfitting.

In addition to the overfitting validation, the 10-fold cross-validation also allowed us to compare the predictive accuracy of different SVMs. For this, the 10-fold cross-validation was repeated and averaged 100 times for each combination of factors in the input. This average value was a metric to quantify the SVM predictive accuracy.

### Data availability

The datasets analyzed during the current study are available from the corresponding author on reasonable request.

## Results

**Figure 2:**
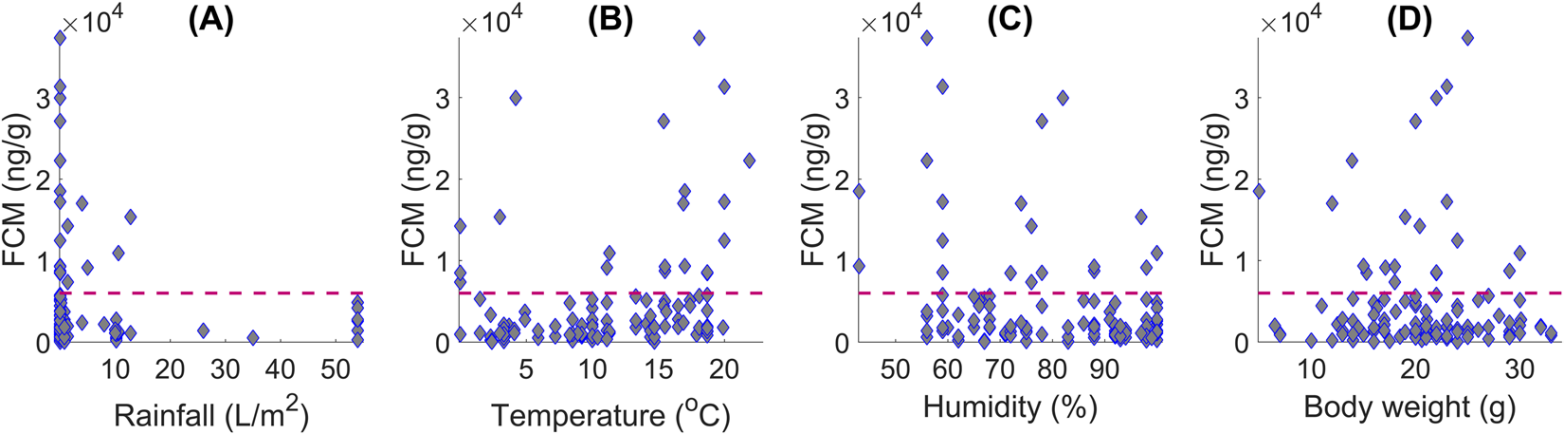
Distribution of the 105 fecal samples analyzed in our study as a function of the four quantitative factors considered as potential stressors: rainfall (**A**), temperature (**B**), relative air humidity (**C**) and body weight (**D**). Dashed line corresponds to a FCM concentration threshold equal to 0.6 ⋅ 10^4^ ng/g.

We first study the correlation between the FCM level in the 105 fresh fecal samples collected during fieldwork and the different environmental and individual factors considered as potential stressors in this investigation. In the case of quantitative factors, we calculate the Pearson correlation coefficient (*r*) with a significance level *p* = 0.05 (Table 2). This measure estimates the linear dependence between two variables. We also generate scatterplots characterizing these relationships (Fig. 2). Our outcomes point out that there is not a strong linear correlation between the FCM level and any of the quantitative factors (the value of *r* lies between −0.31 and 0.28), being statistically significant only the results obtained for temperature and humidity (*p* < 0.01). Moreover, as scatterplots of Fig. 2 illustrate, fecal samples cannot be linearly separated into different categories according to any of these factors and the FCM concentration. Nevertheless, although no general conclusions can be drawn for all the captured mice, it seems that, comparatively, they only presented a high GC level under certain circumstances. The animals showing the highest FCM concentrations were smaller than 25 g, or were trapped when the rainfall level was below 14 l/m^2^ or the temperature was lower than 5 °C or greater than 15 °C. In the case of relative air humidity, samples are distributed more homogeneously.

**Table 2:** Pearson correlation coefficient (*r*) between the FCM level and the quantitative potential environmental and individual stressors considered in our investigation.

	*r*	*p*
Rainfall	−0.11	0.27
Temperature	0.28	0.004
Humidity	−0.31	0.001
Weight	−0.05	0.64

**Figure 3:**
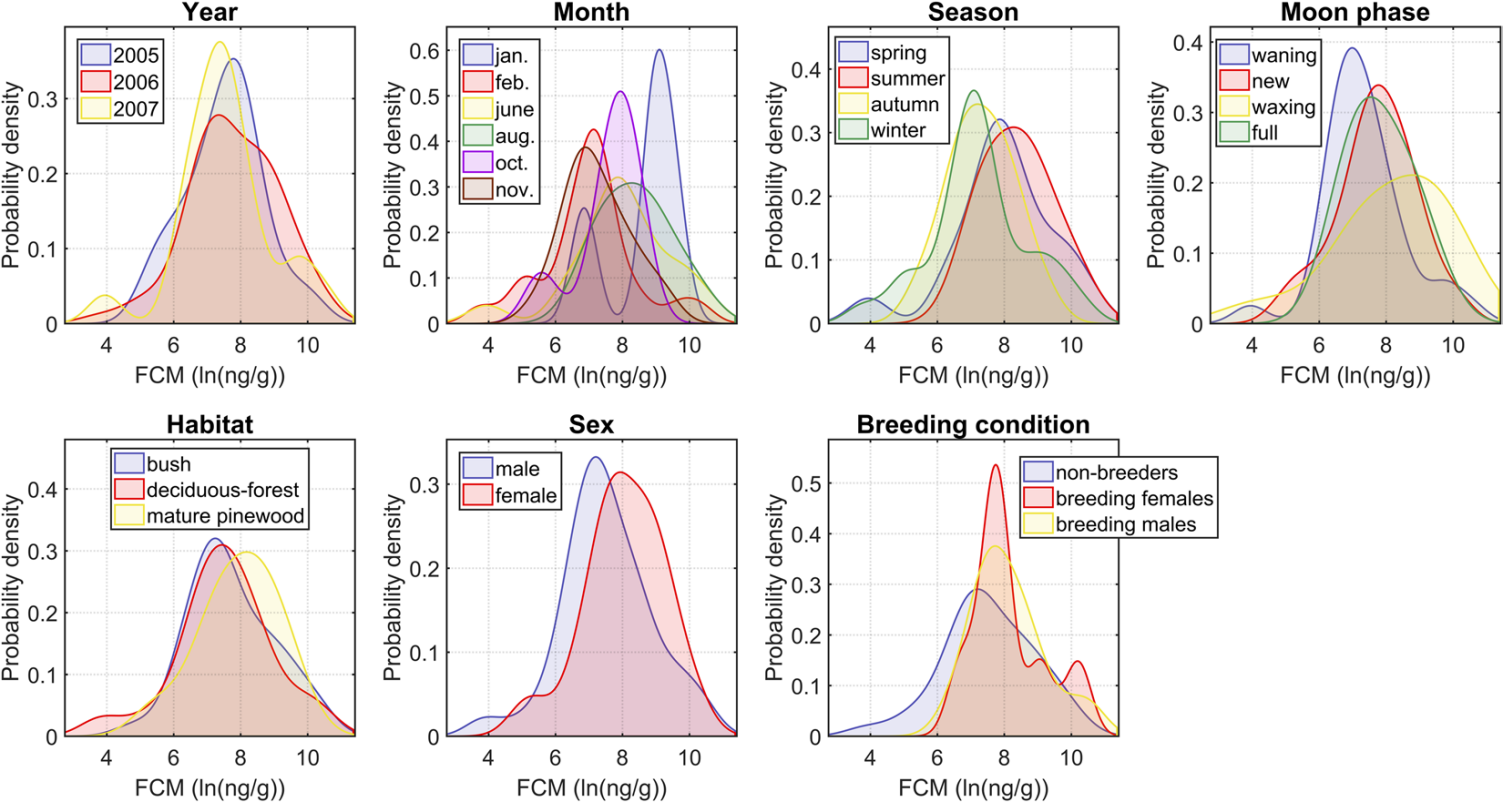
Comparison of the probability distribution of the FCM level according to each possible value of the seven qualitative factors considered as potential stressors in our study. See also Table 3.

Results for qualitative factors are equivalent. In this case, we calculate and compare the probability distributions of the FCM concentration for each possible value (Fig. 3). It can be visually appreciated that, in general, these probability distributions are broad and cover a wide overlapping range of FCM levels. The corresponding means and standard deviations (Table 3) corroborate quantitatively these observations. Then, taking into account the properties of these probability distributions, a unique qualitative factor cannot be used to explain the FCM level observed in all the fecal samples (cf. the probability density maximum peaks in Fig. 3). However, peaks in the probability distributions do allow us to identify some general trends in our data. In this regard, it is important to highlight that some of these trends can be due to the small number of samples. For example, the probability distributions of the two winter months during which we performed fieldwork are clearly different. For January, we observe two peaks. One of them agrees with the main peak in the probability distribution of February, but the main peak for January indicates an increase FCM concentration in this month. However, with only four fecal samples collected in January, we cannot exactly know whether the differences between these two months are because the mice captured in January were mainly outliers, or because GC concentrations are actually higher in January. If we focus on data with enough statistical significance, we observe that:

- During spring and summer, animals generally showed a higher FCM concentration than during winter and autumn.
- Regarding the moon phase, the wood mice with the highest FCM levels were captured during waxing moon. Although, in this regard, it is worth noting the broadness of the probability distribution for waxing moon (cf. maximum peak values in Fig. 3).
- Regarding breeding condition and sex, breeding individuals tended to have an increase FCM concentration as compared to non-breeding animals. While, independently of the breeding condition, the concentration in most females was greater than in males.
- Finally, an interesting outcome is that no significant differences appeared in the stress response of animals living in different habitats.

**Table 3:** Number of fecal samples and mean FCM concentration (ng corticosterone metabolites/g dry feces) per qualitative potential stressor considered in our study.

Parameter	#samples	mean ± SE	SD
Year			
2005	19	3,437 ± 1,151	5,019
2006	65	5,063 ± 843	6,800
2007	21	4,845 ± 1,898	8,699
Month			
Jan.	4	7,755 ± 2,723	5,447
Feb.	24	3,083 ± 1,327	6,499
June	17	6,668 ± 2,320	9,567
Aug.	28	7,223 ± 1,514	8,010
Oct.	7	2,603 ± 590	1,560
Nov.	25	2,292 ± 538	2,689
Season			
Winter	28	3,750 ± 1,225	6,484
Spring	17	6,668 ± 2,320	9,567
Summer	28	7,223 ± 1,514	8,010
Autumn	32	2,360 ± 436	2,467
Habitat			
Mature pinewood	24	4,722 ± 955	4,680
Deciduous forest	43	4,504 ± 1,159	7,603
Bush	38	4,977 ± 1,201	7,406
Moon phase			
New	47	4,117 ± 774	5,307
Waxing	19	8,378 ± 2,203	9,602
Full	8	3,570 ± 1,217	3,443
Waning	31	3,706 ± 1,317	7,333
Sex			
Female	31	5,514 ± 1,088	6,058
Male	74	4,394 ± 843	7,250
Breeding condition			
Non-breeders	77	4,082 ± 643	5,645
Breeding females	7	6,585 ± 3,554	9,403
Breeding males	21	6,464 ± 2,123	9,731

“SE” and “SD” denote standard error and standard deviation, respectively.

**Figure 4:**
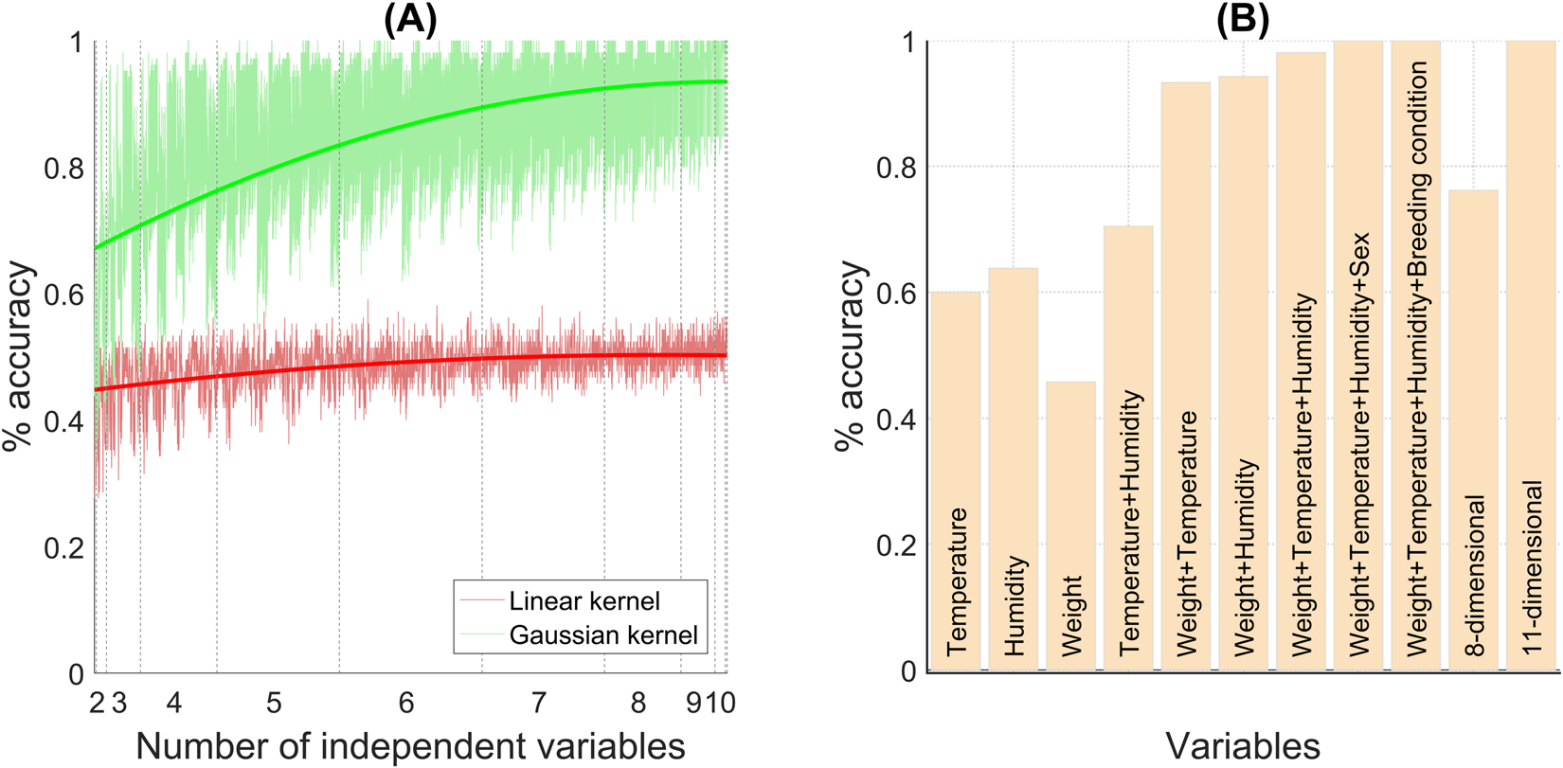
(**A**) Performance comparison of the Gaussian and linear SVM classifiers as a function of the input feature vectors size. Performance is measured as the percentage of fecal samples correctly classified. Note that in this representation we do not distinguish the stressors included in the feature vector, but only the model dimensionality. Dark-solid lines illustrate the global tendency. (**B**) Comparison of some relevant Gaussian classifiers’ performance. Label inside the bar identifies the factors used to build the model. “8-dimensional” correspond to a SVM based on year, month, season, rainfall, habitat, moon phase, sex, and breeding condition; while “11-dimensional” to one based on the 11 environmental and individual factors analyzed in our study.

With the analyses described so far, we have identified the connection among FCM level fluctuations in specific subsets of mice and the value of some environmental and individual factors. However, these findings only explain occurrence of stress in a small percentage of captured animals. This result points out that multiple factors may simultaneously influence the mice physiological stress response, suggesting complex cause-effect relationships between stressors the observed stress response. To elucidate these complex relationships and find out how the combination of different factors can induce a given stress response, we carried out a SVM-based analysis. Figure 4 displays the main outcomes of the brute-force analysis. A first relevant result illustrated in panel A of this figure is the difference of using a linear and a nonlinear approach. On one hand, (nonlinear) Gaussian classifiers have a greater performance than the equivalent linear classifier regardless of the model dimension. On the other hand, linear classifiers show a nearly constant predictive accuracy, while the nonlinear classifiers’ accuracy grows and tends to 100% as the number of dimensions increases (cf. dark solid lines in Fig. 4A). These results are in agreement with the ones previously described, pointing out that our mice fecal samples are not linearly separable in relation to the analyzed stressors, and emphasizing the need of using a nonlinear analysis to explain causality of the stress response.

**Figure 5:**
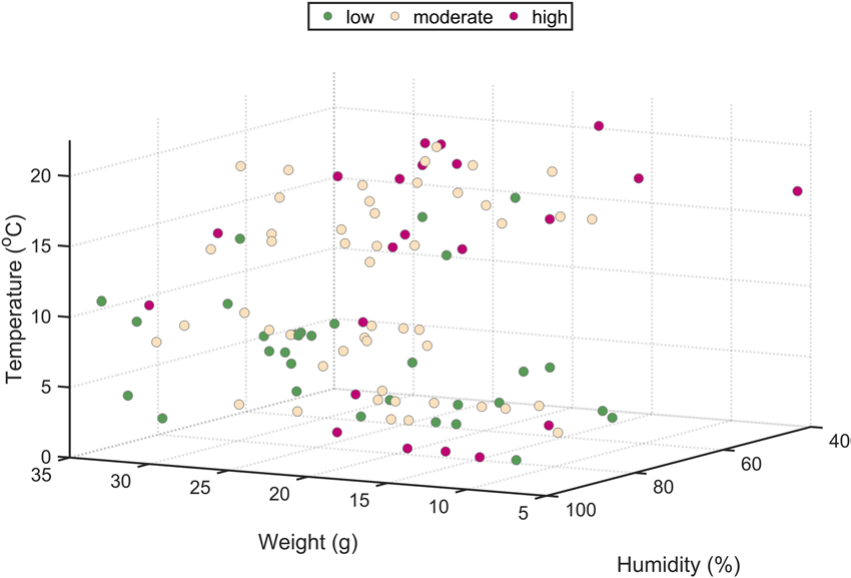
Distribution of the 105 captured mice in a three-dimensional space formed by body weight, temperature and relative air humidity. The color code indicates the level of stress according to the three-class categorization: low, moderate and high (Fig. 1C).

Focusing on the Gaussian models, performance of the one-dimensional SVM classifiers lies between 0.27 and 0.64. Note that in the worst case this is equivalent to performance of a random classifier. These values corroborate the low individual predictive accuracy of factors like year (0.27), rainfall (0.30), breeding condition (0.32) or habitat (0.34), i.e., these factors are weakly correlated with the FCM level. In contrast, SVMs based on relative air humidity or temperature are able to classify correctly 64% and 60% of fecal samples, respectively (Fig. 4B). In other words, individual variations of one of these variables explain around 60% of the fluctuations in the FCM level. Note that these are also the stressors with a stronger linear correlation with the FCM concentration (Table 2). Incorporating additional factors to the models improves outcomes and corroborates the relevance of humidity and temperature for explaining the stress response of the captured mice. In particular, the two-dimensional SVM classifier based on body weight and humidity and the one based on body weight and temperature are able to classify properly 99 and 98 out of 105 fecal samples, respectively (Fig. 4B). Performance of the rest of two-dimensional models, including the SVM that combine humidity and temperature, is below 0.76. These results suggest a strong correlation between the mouse’s weight and its level of stress. However, it is important to keep in mind that the Pearson correlation coefficient between these two variables indicates that they are weakly correlated (*r* = −0.05, Table 2). Similarly, performance of the one-dimensional classifier based on body weight is 0.46. Therefore, the combination of body weight and humidity/temperature is what seems to correlate (nonlinearly) with a specific FCM concentration. Results obtained with more-than-two-dimension classifiers agree with this observation. Multiple combinations of three factors allow predicting the level of stress of more than 90% of captured mice. The best-performer three-dimensional SVM is the one based on body weight, temperature and humidity (with a predictive accuracy of 103 out of 105 samples). Other 16 three-dimensional models have a performance greater than 0.90. All of them are based on body weight and all the possible combinations of temperature or humidity with one of the other eight stressors considered in our study. Similarly, two combinations of four factors show a 100% predictive accuracy: body weight, humidity, temperature and sex/breeding condition (Fig. 4B). Here on, predictive accuracy is 100% for different combinations of factors, but in all cases body weight, humidity and temperature participate in the model. In contrast, the SVM classifiers built not taking into account any of these factors show a lower performance. For instance, the eight-dimensional SVM that includes the other eight potential stressors classifies correctly 71% of fecal samples. These findings point out a strong connection of body weight, humidity and temperature with the stress response in wood mice. Figure 5 illustrates the complex interactions among these three factors to produce a specific stress response in the captured individuals.

**Figure 6:**
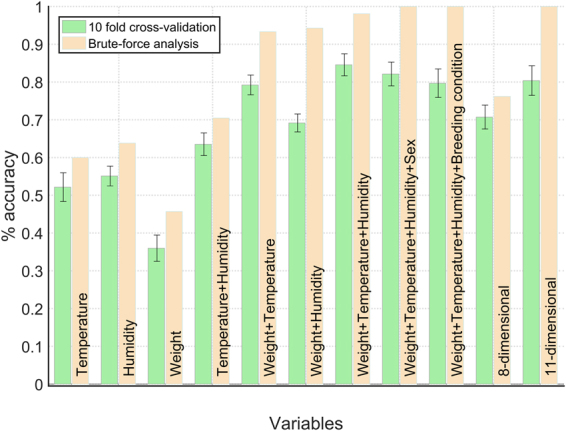
Results of the 10-fold cross-validation and comparison with the results obtained in the brute-force analysis (cf. Fig. 4B). Average data were generated repeating 100 times the 10-fold cross-validations with different random seeds.

Finally, Fig. 6 compares results of the brute-force analysis and the 10-fold cross-validation. As expected, predictive accuracy in the cross-validation is lower, since the testing samples now are not included in the training set. However, the difference between accuracy in the brute-force analysis and in the cross-validation is not very significant, which allows us to be confident that our models are not incurring in overfitting. The general trends described above are kept. Body weight, humidity and temperature are the factors providing better predictions. Furthermore, in the cross-validation, the three-dimensional SVM classifier based on these factors is the best-performer model (0.85 ± 0.03). Then, we can conclude that the 10-fold cross-validation validates the generality of our result, and that body weight, relative air humidity and temperature are the factors that best explain the FCM concentration in the collected mice fecal samples.

## Discussion

Physiological stress response is an adaptive response executed by organisms in order to cope with unpredictable situations^4^. Both environmental and individual factors can act as stressors in wild mammal populations, triggering the physiological stress response^76^. In this investigation, we studied the effect of different potential stressors in 105 wild wood mice captured in Montes do Invernadeiro Natural Park (Galicia, Spain). Physiological stress response of the captured animals was characterized by means of the FCM concentration. We have identified different relationships among some environmental and individual factors and the occurrence of stress in a given animal. Some of these relationships are in agreement and other in conflict with our departing hypotheses. Breeding individuals and females showed a higher level of stress in average than non-breeders and males, respectively (Fig. 3). These observations agree with previous studies on other rodent species and habitats^12,13,50,77^. The request for maternal investment during pregnancy and lactation could explain the increase FCM levels in breeding females. Furthermore, breeding females often experience multiple metabolic changes where GCs play a major role^76,78–80^. In males, the competition for mating females increases aggressive behavior what might explain the elevated FCM concentration in breeding males. The different mean FCM level in females and males agrees with the observation of a different basal GC level between sexes due to differences in metabolism and excretion rate^47,48^. We also observed that the stress response was commonly greater in animals captured in June and August (Fig. 3). This result could be also related to physiological and/or behavioral changes induced by breeding, since the reproductive period in the study area occurs in these months (late spring and summer)^29^. In other cases, we detected that a specific stress level was correlated with the value of a given stressor (Fig. 2). For instance, the highest GC levels occurred for animals captured during a specific moon phase, when the rainfall level is below a given threshold, or for too low or too high temperatures. These findings allow us to understand the response of an average individual to a stressor or even to specific combinations of stressors. Nevertheless, they do not explain the variation of the FCM concentration in all the fecal samples of our dataset because of the broadness and overlapping of the FCM level distributions for all the analyzed factors. This result corroborates that stress response is a complex physiological response where multiple factors interact simultaneously in a nonlinear manner.

To address the study of the underlying mechanisms triggering a specific stress response, we used a SVM-based approach. SVMs present two relevant properties for our study. On one hand, SVM classifiers have the ability to learn complicated decision boundaries when the data present complex distributions. On the other hand, we had a small dataset with a relative large dimensionality of the feature space. This represents an obstacle for many statistical methods, because, in general, the larger the set of available samples, the better the generalization ability^81^. However, SVMs estimate the separating hyperplanes by means of a limited number of training samples characterizing each class (the support vectors). This permits obtaining an optimal classification performance in high-dimensional problems and/or with a low ratio of training samples versus dimensions of the input data (e.g., see refs^82–84^).

The constructed SVMs verified that multiple factors may influence, directly or indirectly, the level of stress in a wild wood mouse, but the ones that best explained the stress response in the 105 captured animals were body weight, relative air humidity and temperature. Classifiers built with only an individual (body weight) and an environmental factor (relative humidity or temperature) were able to predict variations in the FCM levels observed in more than 90% of fecal samples. This predictive accuracy improved combining the three factors. The corresponding three-dimensional model was able to predict the stress response in more than 98% of captured mice. Thus, many of the correlations found in the average FCM levels do not seem to be as significant as one could initially expect. In particular, we would like to highlight the role of seasonality and the dependence of the basal FCM level on sex and breeding condition. Performance of linear and Gaussian three-dimensional classifiers based on these factors were 0.48 and 0.52, respectively (cf. performance of models based on body weight, air humidity and temperature). This does not mean that these factors do not influence the stress response. As we discuss above, it is obvious that they do. However, under some circumstances, stressors with a stronger impact can mask their effect. For instance, multiple climatic factors can fluctuate within a season, which may bias the dependence of the FCM level on seasonality. Indeed, several investigations on *A*. *sylvaticus* have reported a higher food availability during unfavorable climatic seasons^28,85,86^. In other cases, a variable or combination of variables summarizes the role or effect of other stressors. For instance, the trade-off between temperature and humidity better characterize favorable and unfavorable climatic periods than seasonality; and, at the same time, specific combinations of temperature and humidity are correlated with the reproductive period or with a given habitat.

Our results seem to be partially related to the role played by temperature and humidity in primary production. Previous studies have attested the important relationship between climatic conditions and the state of small mammal populations^86–88^. In this sense, temperature and humidity have a significant influence on trophic resource availability and vegetation cover thickness^89–91^. During favorable climatic periods, a large number of seeds, fruits and invertebrates are available for small mammal species like *A*. *sylvaticus*. This food availability produces a body condition improvement in rodents^85,92,93^. In addition, vegetation cover also provides shelter against predators^32,94^. In this manner, an optimal trade-off between temperature and humidity could modify the stress level in a wood mouse in two different ways. Firstly, by increasing the food availability, and hence improving the individuals’ body condition. Secondly, by causing an appropriate growth of the vegetation cover, which involves an improvement in predator avoidance.

**Figure 7:**
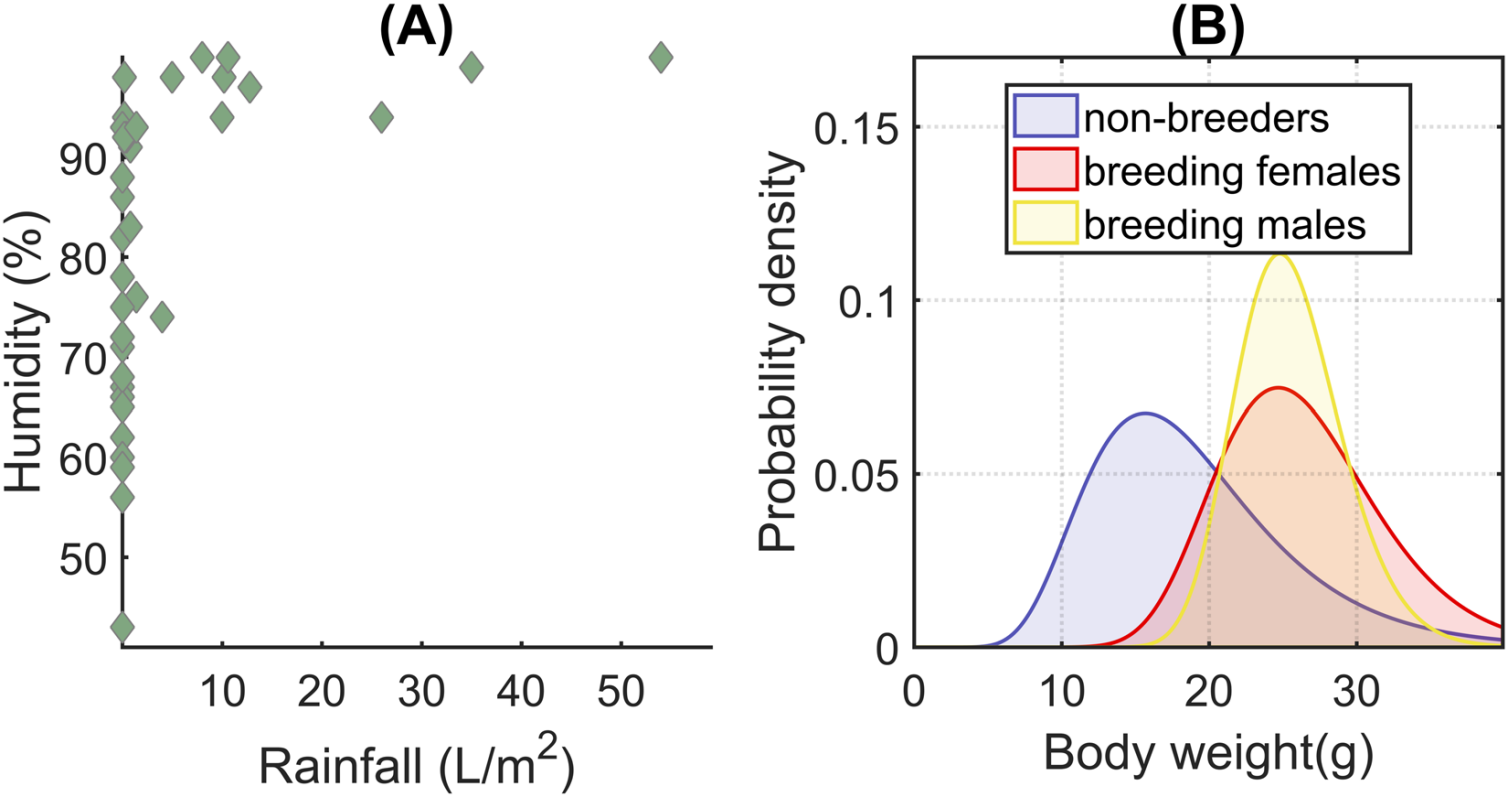
(**A**) Correlation between rainfall and relative air humidity in the study area. The Pearson correlation coefficient between these two variables is *r* = 0.46 with a significance level *p* < 0.001. This means that not in all cases humidity is related to rainfall. Note to what extent high humidity values occurs even without rainfall. (**B**) Probability distribution of the body weight for each possible breeding condition. Breeders are in general heavier than non-breeding individuals.

An interesting result related to weather conditions was the weak correlation between the FCM concentration and the rainfall level as compared with other climatic factors. As temperature and humidity, rainfall significantly influences primary production. During rainy periods, food availability usually grows and the vegetation cover is denser^95–97^. Due to this, rainfall is often considered one of the main environmental stressors for small mammals. Nevertheless, our SVM classifiers did not identify it as a critical factor triggering the stress response. A possible explanation to this result is the weather conditions in the study area. Ambient humidity in Montes do Invernadeiro Natural Park is in general high during the whole year and it is not directly associated with rainfall (Fig. 7A). With this high ambient humidity, this has a similar effect to rainfall on trophic resources and vegetation cover, even during dry periods. This makes humidity a more informative variable. This result could suppose a threat for the generalization of our findings. However, the situation observed in Montes do Invernadeiro Natural Park is a common situation in multiple habitats. Then, we hypothesize that, in general, humidity could be a stressor with a stronger impact on small mammal than rainfall. If humidity correlates with rainfall, both provide the same information; but when humidity has a distinct origin (a nearby water stream, night dew, fog, etc.), it is clearly more informative. Therefore, we argue that humidity should be an environmental factor to take into consideration in small mammals stress studies.

Although at a first glance body weight did not appear to be strongly correlated with the FCM level (see Fig. 2 and Table 2), our SVM models pointed out that it was a highly informative variable. Our interpretation of this result is that the animal’s weight is linked to multiple causes of stress inducing different responses depending on the context. Weight is an indirect measure of body size and condition, so it is closely associated with the ability of individuals to face up the wide range of daily threats. For example, it is supposed that stronger individuals avoid predation more successfully than weaker ones^52,98^. Body weight has also a strong correlation with a wood mouse’s age and, therefore, with its breeding condition (Fig. 7B). Its correlation with age also makes weight a suitable indicator of the social range and behavior of an animal in the population. According to this, it has been reported that subordinate individuals (generally juveniles and sub-adults; 0–20 g^99^) tend to occupy poor quality habitats, i.e., habitats with lower food availability and higher predation risk, due to territorial behavior and intraspecific competitions^100,101^. Territorial exclusion also affects the home range size, and therefore the distance animals have to travel in order to fulfill their energetic demands^44^. Therefore, it seems plausible that younger and smaller individuals exhibited higher concentration of FCM because of a restricted access to resources, and because they probably occupied areas with less dense vegetation cover, which implied poorer body condition and an elevated predation pressure. Our outcomes showed that this negative effect was more evident during periods with extreme climatic conditions – cold temperatures and low relative humidity in our case (cf. red points for less-than-20-gram individuals in Fig. 5). At the same time, heavier individuals are commonly breeders (Fig. 7B). This makes it reasonable to suppose an increased FCM level in these animals during the reproductive period. Thus, many of the captured mice whose weight was greater than 20 g exhibited a high stress level with warm temperatures and an intermediate/low relative humidity. These climatic conditions can be linked up with the reproductive period in the study area (late spring and summer). In contrast, during the rest of the year, these animals showed comparatively lower FCM levels (cf. the concentration of green points in Fig. 5 for more-than-20-gram animals when temperature is below 15 °C). These results emphasize again the complex interaction among stressors to produce a context-dependent physiological stress response.

In brief, our investigation points out that traditional statistical methods may not be enough to fully understand the causality among stressors and the stress response in wild mammals. This could be a common situation in multiple complex biological process, where we often analyze small datasets, with complex distributions and in a large number of interacting dimensions. Our results demonstrate that SVM-based analysis is a reliable statistical approach in this scenario. The SVM models built in this work have allowed us to identify meaningful correlations not found with more traditional analyses. In particular, the animal’s weight and climatic factors such as relative air humidity and temperature appear to be accurate factors to explain and predict the variation of the FCM level in the captured wood mice. Our findings suggest a close correlation between weight and body condition, which seems to have a direct effect on intraspecific relationships, e.g., social dominance and territorial behavior, and the stress response they produce. Temperature and humidity are factors to take into account due to their significant influence in vegetation, being optimal climatic conditions crucial for the survival of small mammals. In contrast, factors traditionally considered the main stressors in small mammals, e.g., sex, breeding condition or seasonality, seem to have a secondary role in the stress response of the wood mice we trapped. One threat to validity of our findings is the small number of samples, mainly in the case of some animal subgroups, e.g., breeding females. One of the expected benefits of using SVMs was their potential generalization ability with a limited number of representative samples (i.e., training samples containing relevant characteristic information). This seems to be the case of our breeding females, since they present patterns previously reported in literature (e.g., higher mean GC levels than males and non-breeding females). Unlike breeding females, other mouse subgroups do not appear to provide representative sample sets. For example, if we compare the GC levels of the four mice captured in January with levels of the ones captured at other winter months, these FCM concentrations seem to mainly represent outlier values. In any case, our results show that the stress response is not highly connected with these stressors. For instance, specific combinations of weather conditions are more informative than month or season to identify favorable and adverse periods linked up with low or high stress responses. In addition, the 10-fold cross-validation empirically corroborated the theoretical ability to generalize of our SVM classifiers (Fig. 6). Therefore, taking into account the above, we conclude that our findings for explaining FCM level fluctuation in Wood mice are valid and general for the study area. Regarding the generalization of the findings beyond our study area, it is important to emphasize that, due to the properties of this region, we did not incorporate to our models some highly relevant potential stressors, e.g., different forms of human disturbance. It is obvious that these factors can play a key role in the small mammals’ stress response depending on the habitat. However, we consider that our main findings are general enough to argue that they would also apply to habitats with a limited human presence. The only difference could be that in habitats with certain climatic conditions the role of rainfall might change and substitute humidity as a key climatic factor. We hypothesize that in habitats with a greater human presence, human disturbances would be additional context-dependent environmental factors interacting with body weight and the key climatic factors. To validate these hypotheses and fully understand the physiology and behavior of small mammal populations, further long-term studies including detailed climatic data of varied climatic regions should be performed.
